# Competition for safe real estate, not food, drives density‐dependent juvenile survival in a large herbivore

**DOI:** 10.1002/ece3.6289

**Published:** 2020-06-09

**Authors:** Mark A. Hurley, Mark Hebblewhite, Jean‐Michel Gaillard

**Affiliations:** ^1^ Idaho Department of Fish and Game Boise ID USA; ^2^ Wildlife Biology Program Department of Ecosystem Sciences and Conservation W.A. Franke College of Forestry and Conservation University of Montana Missoula MT USA; ^3^ Laboratoire Biométrie & Biologie Évolutive CNRSUMR‐CNRS 5558 University Claude Bernard ‐ Lyon I Villeurbanne Cedex France

**Keywords:** habitat selection, ideal despotic distribution, ideal free distribution, predation risk, predator removal experiment, ungulate

## Abstract

Density‐dependent competition for food reduces vital rates, with juvenile survival often the first to decline. A clear prediction of food‐based, density‐dependent competition for large herbivores is decreasing juvenile survival with increasing density. However, competition for enemy‐free space could also be a significant mechanism for density dependence in territorial species. How juvenile survival is predicted to change across density depends critically on the nature of predator–prey dynamics and spatial overlap among predator and prey, especially in multiple‐predator systems. Here, we used a management experiment that reduced densities of a generalist predator, coyotes, and specialist predator, mountain lions, over a 5‐year period to test for spatial density dependence mediated by predation on juvenile mule deer in Idaho, USA. We tested the spatial density‐dependence hypothesis by tracking the fate of 251 juvenile mule deer, estimating cause‐specific mortality, and testing responses to changes in deer density and predator abundance. Overall juvenile mortality did not increase with deer density, but generalist coyote‐caused mortality did, but not when coyote density was reduced experimentally. Mountain lion‐caused mortality did not change with deer density in the reference area in contradiction of the food‐based competition hypothesis, but declined in the treatment area, opposite to the pattern of coyotes. These observations clearly reject the food‐based density‐dependence hypothesis for juvenile mule deer. Instead, our results provide support for the spatial density‐dependence hypothesis that competition for enemy‐free space increases predation by generalist predators on juvenile large herbivores.

## Graphical abstract

We conducted a field experimental test of whether density itself, or, competition for space drove juvenile neonate mule deer survival in Idaho. Taking advantage of an ongoing predator reduction by state agencies, we found that it was competition for space, not density‐dependent food competition per se, that drove juvenile mule deer fawn survival.

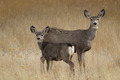

“Many aspects of species’ niches, in ecological and evolutionary time have apparently been molded by interactions with natural enemies for enemy free space. …Yet many ecologists continue to think and write as though classical resource‐based competition for food or space is the primary determinant of species’ niches. Often it is not.”—Jeffries and Lawton (1984)

## 1 INTRODUCTION

Competition for resources is considered the primary mechanism driving density dependence (Bonenfant et al., 2009; Sinclair, 1989), a process especially important to the population dynamics of large mammals (Fowler, 1987). In most species, including large vertebrate herbivores, both pattern (time series of counts) and process(analysis of life history traits in response to change in density) oriented approaches have demonstrated evidence of density dependence as populations approach or exceed nutritional carrying capacity (Gaillard et al., 1997; Bergman, Doherty, White, & Holland, 2015; Gaillard, Festa‐Bianchet, & Yoccoz, 1998). In a review of density dependence in large herbivores, Bonenfant et al. (2009) reported strong evidence for density dependence across large herbivores, where the primary mechanism in most studies was a reduction in the per capita availability of food resources.

Fretwell and Lucas (1970) developed the theory of density‐dependent habitat selection to explain habitat selection in nonterritorial and territorial species, using birds as a biological model. If nonterritorial species are distributed in an ideal free manner, increasing density also reduces the strength of selection for high‐quality patches (space) because of density‐dependent competition for food (Fretwell & Lucas, 1970; McLoughlin, Morris, Fortin, Vander Wal, & Contasti, 2010). Density‐dependent changes in habitat selection thus drive population dynamics through an overall decrease in vital rates in the highest quality habitats because of increasing food competition. A prediction of ideal free habitat selection is that individuals experience similar fitness in different habitats as densities increase because of differential density dependence in the rates of competition in those habitats (Fretwell and Lucas 1970).

In contrast, individuals in despotic species may exclude conspecifics from high‐quality habitats thereby forcing other individuals to occupy lower quality habitats, experiencing differential fitness (Fretwell and Lucas 1970). Under ideal despotic distributions, competition for food is no longer the main mechanism of density‐dependent declines in vital rates. Instead, the main mechanism is hypothesized to be competition for high‐quality habitats or space. A habitat quality is often determined not only by bottom‐up resources, but also, safety from predation (Jeffries & Lawton, 1984). For example, White and Warner (2007) reported that the shape of density‐dependent mortality changed across spatial scales in coral reef fishes, and Andren (1990) demonstrated that space competition for higher quality territories conferred lower nest mortality rates for Eurasian Jays (*Garrulus glandarius*). Case studies in large mammals are scarce, yet Mosser, Fryxell, Eberly, and Packer (2009) showed that territorial African lions (*Panthera leo*) achieved higher reproductive success by selecting higher quality territories and excluding other prides. How the interaction between space and predation shape density‐dependent mortality remains unknown for many large herbivores (Bonenfant et al., 2009; McLoughlin et al., 2010). Whether density dependence might be driven by competition for enemy‐free space has rarely been tested in large mammals.

Nearly four decades ago, Jeffries and Lawton (1984) proposed that competition for enemy‐free space could be as important a mechanism driving density dependence as food‐based competition. Differential vulnerability of life‐history stages to predation provides a potential mechanism for such spatially driven, density‐dependent mortality resulting from competition for enemy‐free space. Across many species, juvenile survival is often the most important vital rate driving variation in population dynamics (Gaillard et al., 1998; White & Warner, 2007). In addition, the juvenile life‐history stage is the most sensitive to density dependence in large mammals (Bonenfant et al., 2009). Juvenile mortality usually increases when breeding females are forced into lower quality habitats (i.e., with lower forage quality and higher risk) as density increases (Fretwell & Lucas, 1970). This source–sink pattern of density‐dependent habitat selection may reduce population productivity as total adult female numbers increase, but critically, would do so unevenly across the population in space. If enemy‐free space is a component of habitat quality, then predation risk could drive density‐dependent mortality (Jeffries & Lawton, 1984).

Juvenile survival drives population dynamics of large herbivores (Gaillard et al., 1998), who remain with their mothers for their first year of their life, and are thus dependent on their mothers’ habitat selection strategies (Shallow, Hurley, Monteith, & Bowyer, 2015). While females in most large herbivores are generally not territorial, some species, such as roe deer (*Capreolus capreolus*), white‐tailed deer (*Odocoileus virginianus*), and mule deer (*O. hemionus*) females are territorial during juvenile rearing in summer (Kjellander, Gaillard, Hewison, & Liberg, 2004; Mackie, Pac, Hamlin, & Ducek, 1998; Ozoga, Verme, & Bienz, 1982). Moreover, older females often select higher quality juvenile‐rearing habitats and experience higher juvenile survival. This is consistent with predictions of ideal despotic distribution, and a prerequisite for competition for space, not food per se, to drive juvenile survival (Ozoga et al., 1982). Thus, a critical prediction of spatial, predation‐mediated density dependence is that territoriality must be evident where dominant individuals exclude conspecifics from high‐quality habitat into low‐quality, higher predation‐risk habitats at increasing densities (Fretwell and Lucas 1970).

The spatial density‐dependent predation hypothesis has rarely been tested, however, especially for large herbivores. Under the food‐based density‐dependence hypothesis, juvenile mortality should increase consistently with density (Bonenfant et al., 2009), irrespective of the type of predator–prey system (single, multiple predators, etc.). To uphold this hypothesis, two processes must be manifested. Alternatively, if competition for enemy‐free space drives density‐dependent survival mediated by predation, then we first predict overall juvenile mortality to not necessarily respond to density as expected under ideal despotic distribution (Fretwell and Lucas 1970). Instead, predictions will depend on the specific dynamics of the predator–prey system for that prey species. In multiple‐predator systems, the relationship between juvenile mortality and density will be determined by both predator specialization (Messier, 1995; Sinclair & Pech, 1996), and spatial overlap between the predators (Northfield et al. 2017). For example, juvenile mortality caused by generalist predators (e.g., a type III functional response) should be density‐dependent but overall predation rate by specialist predators (e.g., type II functional response) need not be density‐dependent (Sinclair & Pech, 1996), especially in multi‐prey systems where the presence of alternative prey for the generalist drives a positive Y‐intercept at low densities of the primary prey (Messier, 1995). Moreover, space can affect predator–prey dynamics (Northfield et al. 2017) because the degree of spatial overlap will mediate density‐dependent predation rates as well (Messier, 1995; Pech, Sinclair, & Newsome, 1995; Sinclair & Pech, 1996). For example, Northfield et al. (2017) demonstrated that very different predation rates emerged in simulations of a two predator–one prey system dependent on the degree of spatial overlap between all 3 species (Northfield et al. 2017).

**FIGURE 1:**
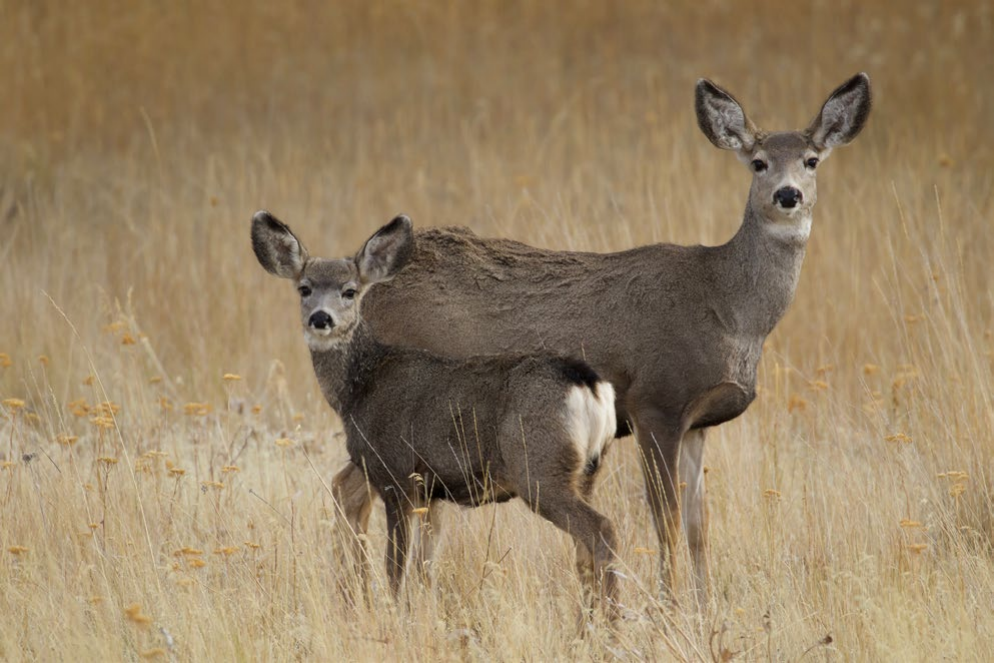
Mule deer female and her juvenile fawn who survived the intense neonatal predation period to make it alive to the fall in Montana, USA. Here, we show competition for enemy‐free space can drive neonatal fawn survival (photo credit: Mark Hebblewhite)

**FIGURE 2:**
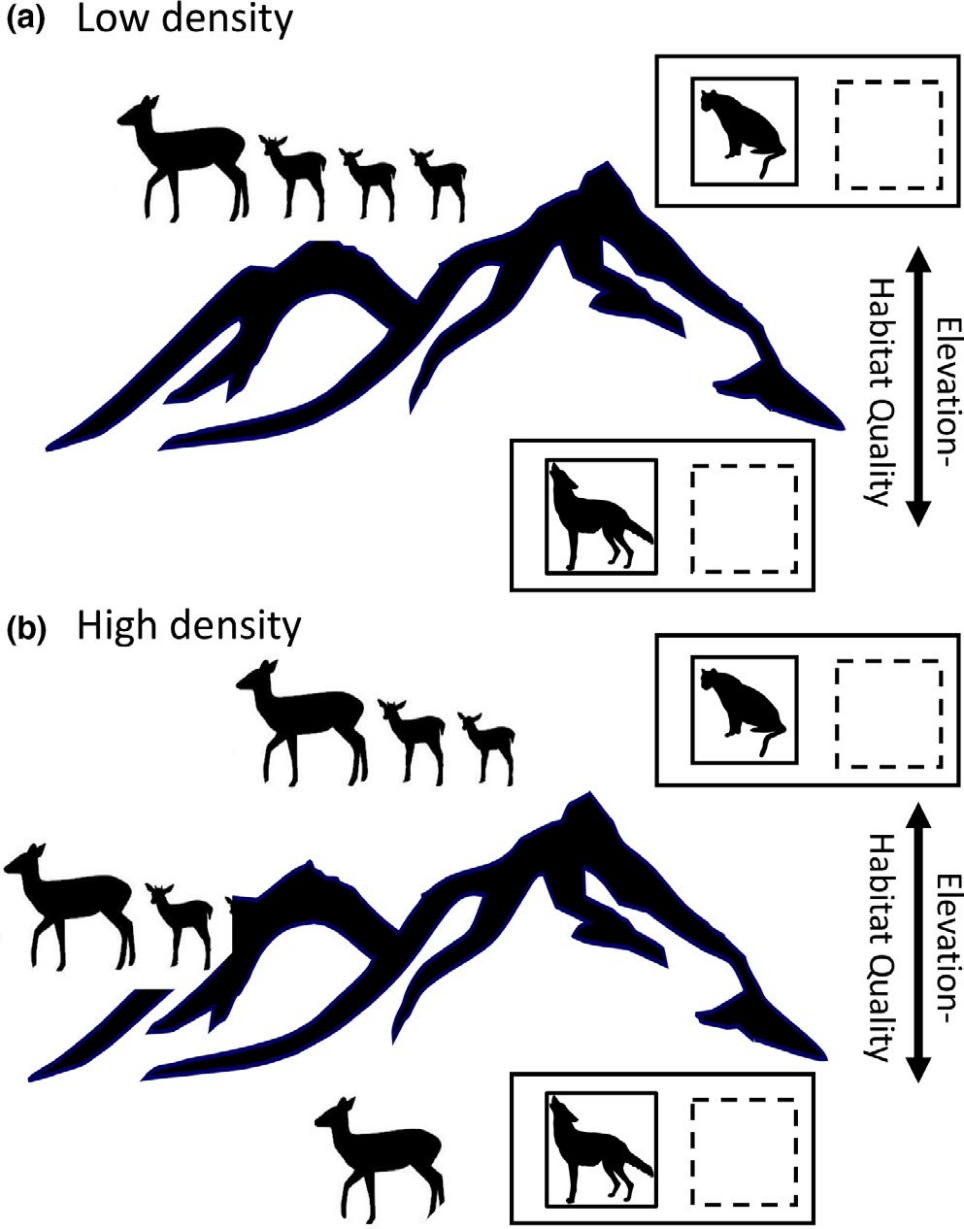
Conceptual figure of our experimental test of the spatial density‐dependent hypothesis mediated by predation on juvenile mule deer (*Odocoileus hemionus*) during summer in southern Idaho, USA. Two adjacent populations had one treatment of coyote (*Canis latrans*) and mountain lion (*Felis concolor*) reduction (dashed box) while another was used as the experimental reference area. In this system, mule deer habitat quality was higher at higher elevations, whereas coyote predation was higher in lower elevation areas, and mountain lion predation was concentrated in high‐quality deer habitat. If competition for low predation‐risk habitats, not food, is responsible for density‐dependent mortality, then there should be no relationship between mortality and abundance overall. Under the spatial predator‐mediated density‐dependence hypothesis, at low mule deer density (a), the highest quality habitat is selected by parturient female mule deer, exposing females and their juveniles to mountain lion but not to coyote predation risk. As mule deer densities increase (b), low elevation ranges with lower forage quality become increasingly occupied, exposing mule deer juveniles to higher coyote mortality, but no changes occur in mountain lion mortality

To fill this knowledge gap, we tested the spatial density‐dependent mortality hypothesis in a predator–prey system with two predators preying on mule deer in southern Idaho, USA (Figure 1). We tested this hypothesis by taking advantage of an experimental reduction of a generalist predator, coyotes (*Canis latrans*) and a specialist, mountain lions (*Felis concolor*) performed over a 5‐year period by Idaho Department of Fish and Game (Hurley et al., 2011). We monitored juvenile survival and cause‐specific mortalities by these two predators for juvenile mule deer for the first 6 months of life in two adjacent subpopulations with and without experimental predator reduction (Hurley et al., 2011). Juvenile survival was the most important vital rate driving population variation (Gaillard et al., 1998; Hurley et al., 2011). Our system was a two predator–one prey system, with 1 specialist predator on deer, and one generalist (e.g., Northfield et al. 2017, Figure 2a). Here, prey overlapped with mountain lions across prey densities, but spatial overlap increased with coyotes only at higher deer densities as subordinate deer expanded into lower quality habitats (corresponding with Figure 2a and predictions in Northfield et al. 2017). Generalist coyotes then switched from their main prey, lagomorphs, to juvenile deer (Patterson, Benjamin, & Messier, 1998). There is a dearth of specific predator–prey theory for two predator–single prey systems, but recent modeling efforts by Northfield et al. (2012, 2017; see Figure a,b therein) and conceptual reviews (Schmitz et al. 2017) support our prediction that differential spatial overlap with 2 predators with increasing density will generate very different expected survival rates and abundances of shared prey. Moreover, while these previous modeling efforts did not explicitly address density dependence, the predator reduction allowed us to experimentally isolate effects of predation from density.

First, we tested for age‐dependent differences in selection for fawn (juvenile)‐rearing home ranges by mule deer females to confirm evidence for territoriality with respect to exposure to predation risk (Mackie et al., 1998; Ozoga et al., 1982). We predicted that dominant individuals will be older as defined by the matriarchal social organization of deer (Ozoga et al., 1982), therefore occupying the highest quality habitat, similar to emerging evidence from other cervids (Froy et al., 2018; Nussey et al., 2007). In our system in semi‐arid Idaho, the highest quality summer habitat occurred at higher elevations in mesic aspen and shrubland communities (Figure 2, Stoner et al. 2018). Thus, we predicted older females would occupy high‐quality juvenile‐rearing habitat more than younger females.

Second, we compared juvenile survival in response to manipulated predator density and natural variation in mule deer abundance to test predictions of the density‐dependent predation hypothesis in our specific predator–prey system (Table 1, Figure 2). In our system, spatial separation of the generalist, coyote, and specialist, mountain lion occurred across an elevational gradient that also corresponded to a forage quality gradient where mule deer forage quality was highest at higher elevations (Figure 2, Stoner et al., 2018). Coyotes were a generalist predator whose densities and habitats were strongly tied to their lower elevation dwelling, primary lagomorph prey (Hurley et al., 2011; Patterson et al., 1998). Therefore, coyote predation on mule deer juveniles occurred more at lower elevations in lower quality mule deer summer habitats and then only at higher deer density (Figure 2, Mahoney et al. 2018; Stoner et al., 2018). In contrast, like African lions, mountain lions are specialist predators that primarily select high‐quality mule deer habitat (higher elevations) in summer (Figure 2; Robinson et al., 2015; Stoner et al., 2018). To test whether juvenile mortality increased at high deer density in response to exposure to higher coyote predation risk as deer expanded into lower elevation space, we first developed a spatial model for coyote predation risk on mule deer (e.g., Hebblewhite, Merrill, & McDonald, 2005). We then tested the prediction that higher coyote predation risk was correlated with increasing juvenile mortality (survival, Table 1) as deer density increased using Cox‐proportional hazards models. In contrast, the predation rate of mountain lions on juvenile mule deer should not increase with deer density if mule deer expand into lower quality, lower elevation deer habitats avoided by mountain lions (e.g., Mountain lion‐free space; Atwood, Gese, & Kunkel, 2009; Stoner et al., 2018; Table 1; Figure 2). This is because the numeric response of higher elevation mountain lions is tied to higher elevation, higher quality mule deer density (Table 1, Figure 2). Thus, mountain lion predation rates on juvenile mule deer are predicted to remain constant as overall mule deer density increased because territoriality of deer means that such density increases occurred only in lower quality, coyote areas at low elevation (Northfield et al. 2017; Schmitz et al. 2017).

**TABLE 1:** Distinguishing food from spatial predation‐mediated density dependence. Hypotheses and predictions for testing current food‐based density dependence in large mammals versus predator‐mediated density dependence for mule deer (*Odocoileus hemionus*) during summer in Idaho, USA. Specific predictions for changes in mortality type under both hypotheses, and, in our predator removal treatments, are provided that help distinguish the two mechanisms for density dependence in our mule deer–coyote–mountain lion system (see Figure 2)

Mortality type	Food limitation‐based density dependence	Spatial predator‐mediated density dependence
Overall Mortality	Should increase with mule deer density after a density threshold is reached due to food limitation	No relationship should occur with increasing density because mortality agents counteract effects, and food is not limited. Predator removal should decrease mortality.
Coyote‐caused mortality	Should decrease with increasing mule deer density in the reference area—coyotes limited by territory and deer limited by food.	Should increase at higher density as mule deer occupy lower quality habitat with predation risk by coyotes in the reference area.
	Stronger decrease with increasing mule deer density in the removal area	Should increase but with a weaker relationship with density because coyote‐caused mortality and mule deer density because fewer predators in the removal area
Mountain lion‐caused mortality	Should decrease with increasing mule deer density in the reference area—mountain lions limited by territory and deer limited by food.	No change should occur in mortality rate with increasing density because deer and mountain lions both utilize lower quality habitat in the reference area.
	Stronger decrease with increasing mule deer density in the removal area.	Should decrease with increasing density because fewer predators in the removal area

Finally, we used the experimental reduction in both predators to separate the effects of density and predation risk from coyotes (low elevation) versus mountain lions (high elevation). We predicted overall juvenile mortality, but not necessarily coyote‐caused mortality, to decline with increasing deer density in the predator removal situation in the case where spatial density dependence would have driven juvenile mortality (Table 1, Figure 2). If our system was driven by the null hypothesis, food‐based density dependence, we would instead predict overall juvenile mortality to increase as other forms of mortality related to food limitation would increase to compensate for the reduction in predator mortality. In contrast, under the density‐dependent predation hypothesis, we predicted strong density‐dependent responses of juvenile mortality caused by coyotes in the reference population. Finally, as predicted by predator–prey modeling (Messier, 1995; Pech et al., 1995; Sinclair et al., 1998) and two‐predator–one‐prey models (Northfield et al. 2017), we predicted that when mountain lions were reduced, we should observe a density‐dependent decline in mountain lion predation, but not in the reference area because deer territoriality limits the increase of overall deer density within high‐elevation habitats following source–sink dynamics predicted by ideal despotic distribution (Figure 2, Table 1; Mosser et al., 2009; Northfield et al. 2017; Stoner et al., 2018).

## 2 MATERIALS AND METHODS

### 2.1 Data collection

We monitored mule deer population size and juvenile survival in Game Management Units (GMU) 56 (2,338 km^2^) and 73A (1,128 km^2^) for 5 years from 1998 to 2002 in southeastern Idaho (described in full by Hurley et al., 2014; Hurley et al., 2017). Elevations ranged from 1,350 to 2,666 m. Each GMU encompassed one entire mountain range and provided suitable yearlong habitat for a distinct subpopulation of deer with minimal interchange between the two GMUs. We took advantage of a concurrent management experiment conducted by Idaho Department of Fish and Game (IDFG) where coyotes and mountain lions were actively removed from GMU 73A (herein after "removal area") and GMU 56 was designated as the "reference area". Coyote removal was conducted by United States Department of Agriculture (USDA) Wildlife Services personnel under their authority and federal permits. The removal of mountain lion was conducted by licensed mountain lion hunters operating under increased hunting permits provided by IDFG (Hurley et al., 2011). A detailed description of study areas and coyote removal treatments, as well as permitting and approvals therein, is provided in Hurley et al. (2011).

Obtaining reliable measures of deer density is critical when testing for density dependence, and ideally, one should either experimentally manipulate density or exploit natural variation in density. Here, we experimentally manipulated predator abundance, but took advantage of natural variation in mule deer density that varied by a factor of 1.67 (see Results). We used aerial surveys via a Bell 47 helicopter to survey the entire winter range and estimate mule deer population abundance following aerial survey methods detailed in Hurley et al. (2011) from late March to mid‐April each year from 1997 to 2003.

To test for age‐dependent differences in juvenile‐rearing home ranges, we captured female mule deer and estimated ages through tooth wear and replacement methodology in the winter of 1998 (Robinette, Jones, Rogers, & Gashwiler, 1957). These females were fitted with radio collars (Lotek LMRT‐3) and located via aerial telemetry on 2–4 occasions during the subsequent juvenile‐rearing season (25 May to 1 August) to estimate female space use. We captured neonates from 28 May to 18 June fitted them with brown or black expandable radio collars designed to break away 6–8 months after capture (see Hurley et al., 2011). Animal capture and handling protocols were approved by the Idaho Department of Fish and Game, and University of Montana IACUC (protocol #02‐11MHCFC‐031811). We monitored telemetry signals for mortality of juvenile deer via aerial or ground telemetry at 1‐ to 2‐day intervals during summer and twice weekly throughout early winter (30 November). We identified the cause of death within 24 hr using criteria developed by Wade and Bowns (1982), and categorized mortalities as caused by coyotes, mountain lions, bobcats (*Lynx rufus*), unknown predators, malnutrition, natural factors, other factors, and unknown.

We developed a spatial model of coyote predation risk using a resource selection probability function (RSPF, Manly et al., 2002; Hebblewhite et al., 2005). We did not construct a mountain lion RSF because they are well known to select high quality, higher elevation deer habitats in our semi‐arid system (Robinson et al., 2015; Stoner et al., 2018). Moreover, the spatial density‐dependent mortality hypothesis predicts no changes in cougar‐caused mortality under either changing coyote or deer abundance (Pech et al., 1995, Table 1). We conducted coyote scat surveys annually to estimate coyote presence or absence (e.g., Mills & Knowlton, 1991). Eighty 1.6 km transects were randomly selected and surveyed across a wider range of GMU’s than just our two focal areas during May to June 1998–2002 (Figure S1), the beginning of the temporal window for most juvenile mortality (Shallow et al., 2015). Landcover types were defined from the SAGEMAP vegetation (Hurley et al. 2017). We placed a 1,000‐m buffer around coyote transects and intersected the resulting polygon with the cover type and spatial covariates from digital elevation models using a Geographical Information System (ArcGIS ver. 9.3.1, ESRI Inc. 2009). We then measured the proportion of each landcover type and other spatial terrain covariates (see below) within the buffer to develop the coyote risk model, which we then mapped across the study area.

### 2.2 Statistical analysis

First, we estimated the relationship between elevation, a key driver of our coyote predation‐risk model (see Results), and female mule deer age to confirm the prediction that older females selected “safer” (e.g., higher elevation) juvenile‐rearing home ranges during summer. We calculated the mean elevation of locations obtained between 25 May and 1 August to test our prediction that dominant older females will exclude subdominant females (younger) from the highest quality habitats located at higher elevations.

We estimated juvenile survival of mule deer in each year using nonparametric Kaplan–Meier survival estimation with staggered left and right entry/exit using the survival package in R (Hosmer & Lemeshow, 1999; Kaplan & Meier, 1958; Therneau, 2019). We used estimated birth date as beginning at risk time (origin) and then entered the analysis on capture date (when individuals entered the risk set), and end time at death or censored from shed collar or end of study (30 November; see Hurley et al., 2011). Next, we estimated cause‐specific mortality rates using cumulative incidence functions in a competing risks format using R code from Heisey and Patterson (2006).

Second, we estimated coyote predation risk using a used–unused resource selection probability function (RSPF) design (Manly et al., 2002). The sample unit was the coyote transect, and the dependent variable, presence or absence, was modeled using logistic regression (Hosmer & Lemeshow, 2000) in the R package lme4 (Bates et al., 2019). This approach assumes predation risk is driven by the relative abundance of coyotes, borne out in studies of other canids preying on ungulates (e.g., Hebblewhite & Merrill, 2007). Coyote scat transects were conducted in both the reference (GMU 56) and removal (73A) areas, as well as neighboring GMU’s (Hurley et al., 2011). We treated year as a random effect to control for year‐to‐year variance in coyote use of transects and nonindependence of repeated trials of transects each year (Gillies et al., 2006). We developed coyote predation‐risk models based on the landcover model, a digital model for elevation, and a measure of terrain ruggedness (Sappington, Longshore, & Thompson, 2007). We conducted model selection using forward and backward stepwise variable selection using AIC_c_ (Burnham & Anderson, 1998). We validated the top coyote RSPF model with k‐folds cross validation (Boyce, Vernier, Nielsen, & Schmiegelow, 2002).

We tested our predictions (Table 1) by including coyote predation risk and deer density in Cox‐proportional hazards survival models using the survival package (Hosmer & Lemeshow, 1999; Therneau, 2019). We measured probability of presence of coyotes from our predation‐risk model at the neonate capture site buffered by 500 m (based on mule deer fawn movements in the dominant mortality period, Hurley, M., *unpubl. data*). We tested the food‐based prediction that density would drive juvenile survival using deer abundance over the preceding biological year (i.e., previous spring survey) as a covariate. Second, we next tested the relationship between the cause‐specific mortality rate of juveniles killed by all mortality causes, by coyotes, and then by mountain lions against mule deer abundance in the combined study areas, and, separated by both the predator removal (GMU 73) and reference (GMU 56) areas to test the hypothesis of a coyote predation‐mediated, spatial density dependence in mule deer (Table 1).

## 3 RESULTS

**FIGURE 3:**
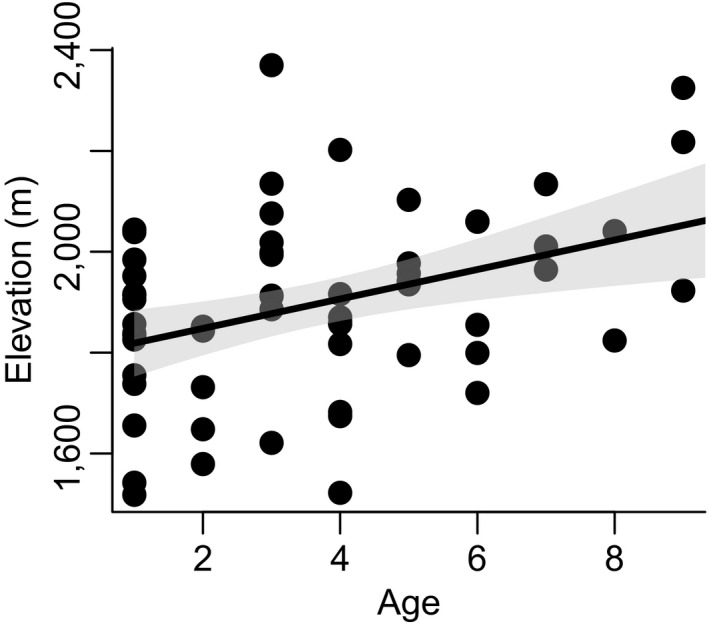
Relationship between the age of female mule deer (*n* = 61) and the elevation (and hence, safety from predation from our generalist predator, coyotes, which was highest at low elevations) of home ranges during late spring and summer in southern Idaho, USA. The gray shading indicates the 95% confidence interval for the effect of age on elevation

Mule deer population size fluctuated by 1.67 over the study period, increasing from 2,810 (reference area, GMU 56 = 1878, removal area, 73A = 932) in 1998 to 4,695 in 2001 (reference = 2,932, removal = 1763) and then decreasing to 3,067 (reference = 1,496, removal = 1571) in 2002 due to severe climatic conditions (see Hurley et al., 2011). We captured and estimated ages for 61 female mule deer in the winter of 1998. In support of our prediction that maternal age would be negative correlated with coyote predation risk, maternal age and elevation (a key driver of coyote predation risk, see below) were positively associated (Figure 3; *β* = 29.22, *F*_1,59_ = 9.92, *p* = .003, *R* ^2^ =.14).

We captured 251 newborn juveniles from 1998 to 2002, a median of 58 year (sample sizes in the control and treatment areas for each year of the study were, respectively, year 1 ‐ 8, 12; year 2 ‐ 20, 29; year 3‐ 32, 30; year 4 ‐ 30, 31; year 5 ‐ 28, 30). Cause‐specific mortality rates in the reference area and in the removal area were, respectively, 0.13 (95% CI 0.075–0.195) and 0.11 (0.059–0.17) due to coyotes, 0.11 (0.061–0.173) and 0.07 (0.027–0.148) due to mountain lions, and 0.08 and 0.05 due to other species or unknown predation (see Hurley et al., 2011). Coyotes were the leading cause of mortality, and the predator removal experiment reduced overall juvenile mortality by 25% in some years (see Hurley et al., 2011). Furthermore, overall mortality rates were higher in low elevation habitats (Hurley, *unpubl. data*). Overall juvenile survival rates varied from 0.20 to 0.74, consistent with previous studies showing wide variation in juvenile mule deer survival.

Coyote scats were recorded on 527 of 1,035 transects with which we developed the coyote predation‐risk RSPF. The random intercept model had a lower AIC_c_ than the best logistic regression without random effects (ΔAIC_c_ = 25.5) or the full model (ΔAIC_c_ = 34.2). Coyotes selected lower elevations and mesic sage habitats and avoided high‐elevation and aspen habitats (Table 2, Figure S1). The top model cross‐validated quite well fivefold cross validation, which yielded a mean *r_s_* = .829.

**TABLE 2:** Parameter estimates of the top coyote (*Canis latrans*) resource use model in southern Idaho, 1998–2002, used to estimate exposure of juvenile mule deer (*Ocodoileus hemionus*) to coyote predation risk, showing the Beta coefficient, z‐score, *p*‐value, and 95% confidence interval (CI)

Vegetation type	Coefficient	*z*	*p*> |*z*|	95% CI	
Intercept	2.48	2.75	.006	0.714	4.25
Aspen	−2.48	−2.54	.011	−4.40	−0.569
Other Deciduous	−3.59	−1.33	.182	−8.87	1.688
Elevation	−0.002	−2.83	.005	−0.002	−0.0005
Mesic sage	0.870	4.02	<.005	0.446	1.294
Mesic grass	−50.3	−2.11	.035	−96.88	−3.65
Developed	6.88	1.99	.046	0.117	13.63

Mule deer juvenile mortality increased with higher coyote predation risk (*β* = 0.938, *χ* ^2^ = 4.0, *p* =.045). The hazard ratio for the effect of coyote presence on juvenile mortality was positive indicating 2.56 higher odds of mortality with increasing coyote presence (95% CI = 0.993–6.58). Though the odds ratio marginally overlapped 1 (no effect), the magnitude of the biological effect was that juveniles with a 100% probability of coyote presence would die 2.56 faster than juveniles born in areas where coyotes were absent. Applying this hazard ratio to the range of predicted probability of coyote presence in our study (0.014–0.72) showed that juvenile mortality would increase about twofold in areas selected by coyotes.

**FIGURE 4:**
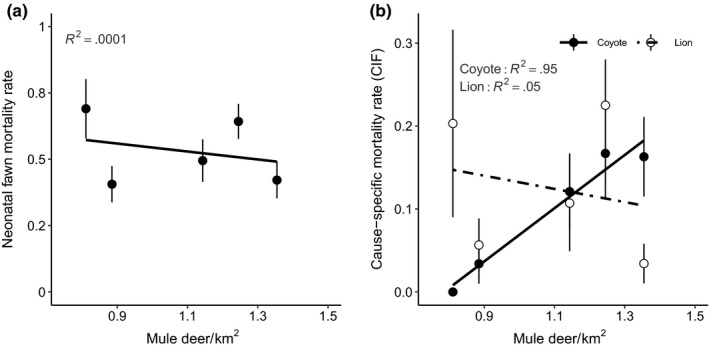
Relationship between mule deer (*Ocodoileus hemionus*) density (deer/km^2^) and (a) overall juvenile mortality and (b) juvenile mortality caused by coyotes (*Canis latrans*) and by mountain lion (*Felis concolor*) including average density and mortality combined across both reference and removal areas (GMU 56, 73A) in southern Idaho, 1998–2002

Contrary to the classic food‐based prediction of density dependence (Table 1), there was no relationship between juvenile mortality and deer abundance across both GMU’s pooled (Figure 4a; *β* = .008, *F*_1,3_ = 0.001 *p* = .98, *R* ^2^ = .0001). Alternatively, the prediction of predator‐mediated density dependence was clearly supported as coyote‐caused mortality increased strongly with mule deer abundance when the 2 GMUs were treated as one population (Figure 4b; *β* = 0.321, *F*_1,3_ = 76.43, *p* = .003, *R* ^2^ = .95). Juvenile mortality caused by mountain lions showed no relationship, suggesting that the overall relationship was driven by increased mortality caused by coyotes (Figure 4b; *β* = −0.080, *F*_1,3_ = 0.147, *p* = .727, *R* ^2^ = .05).

**FIGURE 5:**
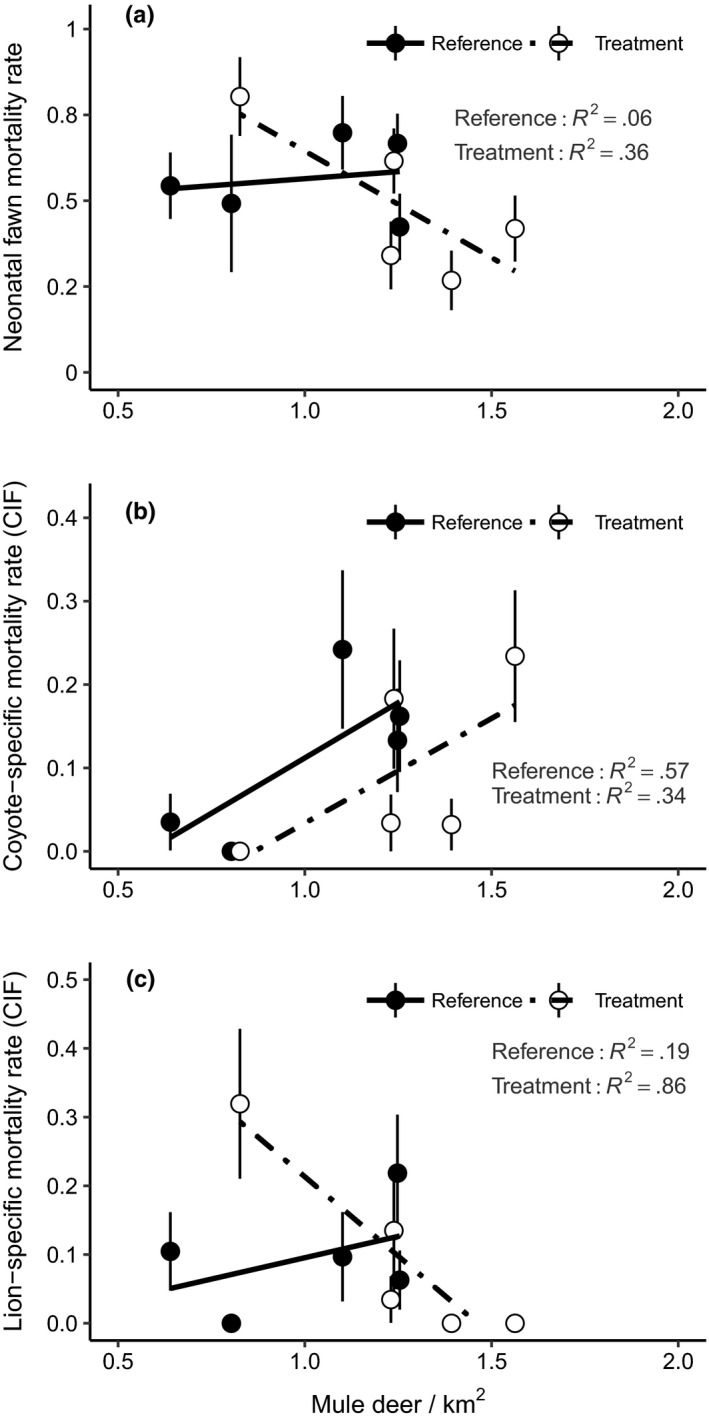
Relationship between mule deer (*Ocodoileus hemionus*) density (deer/km^2^) and (a) overall juvenile mortality, (b) juvenile mortality caused by coyotes (*Canis latrans*), and (c) juvenile mortality caused by mountain lion (*Felis concolor*) in 2 Game Management Units (GMU 56, 73A) that received a predator removal (treatment) and an adjacent control (reference) in southern Idaho, 1998–2002

The prediction of the classic food‐based density dependence hypothesis was also not evident when the GMUs were separated in the reference area (GMU 56, *β* = 0.11, *F*_1,3_ = 0.19, *p* = .69, *R* ^2^ = .06, Figure 5a). In contrast, a negative, but weak, relationship occurred in the removal area (GMU 73A, *β* = −0.540, *F*_1,3_ = 1.67 *p* = .29, *R* ^2^ = .36, Figure 5a), indicating overall juvenile mortality declined with increased density in the predator removal area.

The second prediction of density‐dependent predation risk was borne out when GMUs were separated (Table 1). Coyotes killed more juveniles with increasing population density, even though the overall juvenile mortality was constant or slightly decreasing (Figure 5a). The relationship was strongest in the reference area (*β* = 0.267, F_1,3_ = 3.90, *p* = .14, *R* ^2^ = .57; Figure 5b), but still positive in the predator removal area (*β* = 0.185, *F*_1,3_ = 1.55, *p* = .30, *R* ^2^ = .34; Figure 5b). The pattern was different for mortality caused by mountain lion and consistent with the trend of decreasing overall juvenile mortality with increasing population density (Figure 5a). No relationship was observed in the reference area (*β* = 0.124, *F*_1,3_ = 0.68, *p* = .47, *R* ^2^ = .19; Figure 5c), but there was a strong negative relationship in the predator removal area (*β* = −0.461, *F*_1,3_ = 18.85, *p* = .02, *R* ^2^ = .86; Figure 5c).

## 4 DISCUSSION

Our experimental study clearly rejected the classic food‐based density‐dependence hypothesis for juvenile mule deer survival, and instead, suggests that density‐dependent survival of juvenile mule deer was mediated by competition for safe space, not food per se. As abundance increased, safe space in high quality, higher elevation territories diminished for maternal mule deer, increasing exposure of their juveniles to predation risk by coyotes as mule deer density increased in lower elevation, higher coyote risk areas (Figure 2). In our system, generalist coyote predation was highest at lower elevations, and coyotes caused most juvenile mortality. Accordingly, older female mule deer selected for juvenile‐rearing home ranges at higher elevations (Figure 3), consistent with both predictions of territoriality under the ideal despotic distribution (Fretwell & Lucas, 1970) and results from a growing number of studies demonstrating age‐specific habitat selection by cervids for high‐quality habitats (Froy et al., 2018; Nussey et al., 2007). Juvenile mortality caused by coyotes increased with deer density, but, contrary to what is expected from the food‐based density dependence hypothesis, the overall mortality did not increase with abundance (e.g., Figure 3a,b). Moreover, juvenile mortality caused by a specialist predator, the mountain lion, did not increase with mule deer density (Figure 3b). These results support the hypothesis that female mule deer follow an ideal despotic habitat selection during juvenile rearing, which exposed juveniles to higher predation risk to coyotes at high deer densities. More generally, our results support the importance of predation alone (Jeffries & Lawton, 1984), as well as the trade‐offs between risk and forage in driving density dependence in large herbivores (Hebblewhite & Merrill, 2011; Sinclair & Arcese, 1995).

While female large herbivores are generally not considered as being territorial, there are a number of species and specific life‐history stages or seasons in which competition for space may drive density dependence. Like mule deer (Mackie et al., 1998), roe deer females are solitary when they are raising their juveniles (Kjellander et al., 2004). Ozoga et al. (1982) demonstrated similar territoriality during juvenile‐rearing periods in white‐tailed deer. In white‐tailed deer, females were strongly territorial during neonate rearing, and Ozoga et al. (1982) hypothesized that crowding on juvenile‐rearing ranges increased mortality, especially for younger females (Ozoga & Verme, 1986). While these studies in roe deer and white‐tailed deer did not investigate cause‐specific mortality and were conducted in study sites with no (white‐tailed deer) or low (roe deer) predation on juveniles, most juvenile mortality in large herbivores arises from predation (Griffin et al., 2011; Linnell, Aanes, & Andersen, 1995). Our results showed older females preferred higher elevation areas with lower predation risk by coyotes for juveniles. Thus, variation in predation risk within female territories during juvenile rearing is a likely mechanism driving density dependence in many other vertebrate herbivores.

Our results are also consistent with mule deer selecting lower predation‐risk habitats at low density. Previous studies have shown that mule deer select highly productive aspen (*Populus* spp.) vegetation communities for juvenile rearing in direct contrast to our coyote resource selection (Atwood et al., 2009). Thus, in our system, mule deer females did not face a risk‐forage trade‐off because higher quality forage occurred at higher elevations (Stoner et al., 2018) with lower coyote predation risk, which allowed females to select high forage and avoid coyote predation at the same time (Pierce, Bleich, Monteith, & Bowyer, 2012). Undoubtedly, however, high‐elevation females faced trade‐offs within juvenile‐rearing habitat to avoid predation risk from mountain lions, which may have mediated some compensatory mortality (e.g., Northfield et al. 2017). Regardless, our results are in line with Byers (1997)'s study of a pronghorn (*Antilocapra antilocapra*) population subjected to high coyote predation where maternal habitat selection was driven by antipredator behaviors. Byers (1997) also showed strong maternal age effects on juvenile survival where mothers older than 7 years were better able to select “safe” areas for juvenile rearing. Thus, for large herbivores, predation by generalist predators may drive safe juvenile‐rearing habitat selection, and older mothers are probably better at selecting such safe habitats and outcompeting younger conspecifics.

In contrast to predation by coyotes, predation by mountain lions was highest on high elevation‐high‐quality mule deer juvenile‐rearing habitat irrespective of experimental changes in mountain lion abundance or fluctuations in deer abundance (Hurley et al., 2011; Stoner et al., 2018). This pattern matches recent studies of African lions showing that lions selected the highest quality areas for preying on large ungulate prey (Mosser et al., 2009). Several previous studies in the intermountain west have reported opposing canid and mountain lion habitat selection patterns (Atwood et al., 2009). In general, mountain lions select for high‐elevation areas, rugged, treed‐terrain, in productive, mesic habitats in mountain vegetation communities (Atwood et al., 2009; Robinson et al., 2015; Stoner et al., 2018). In contrast, lower elevation areas in our study dominated by coyote predation risk were sagebrush steppe vegetation communities, shown across studies to be avoided by mountain lions and preferred by coyotes because of the higher abundance of lagomorph primary prey (e.g., Atwood et al., 2009; Patterson et al., 1998; Stoner et al., 2018). As predicted, we did not detect any change in mountain lion mortality with increasing mule deer density. In the reference area, as mule deer increased, there was only a weak increasing trend of cause‐specific mortality by mountain lions (*R* ^2^ = .19, Figure 5c). In the removal area, juvenile mortality caused by mountain lion decreased with increasing mule deer density, which was predicted if mule deer mostly increased in lower quality habitats with higher coyote predation risk (Table 1, Figure 2, 1, 2 al., 1995; Sinclair et al., 1998). In our system, predation by a specialist predator, mountain lions, did not lead to strong density‐dependent predation pressure because territoriality of deer limited expanding deer density into lower elevation, higher coyote predation‐risk habitats. In our system, the existence of a generalist predator, coyotes, that depended on primary lagomorph prey at lower elevations drove these source–sink dynamics. One of the challenges of alternative hypotheses to food‐based density dependence is that specific predictions of the nature of density‐dependent predation will vary across systems, dependent on the nature of predator–prey dynamics and spatial overlap, as exemplified by our study.

Mule deer females likely faced a mountain lion predation risk‐foraging trade‐off at all densities, in contrast to coyotes, for high‐quality resources. Although aspen was a rare cover type in our study area (only 5% of the total landscape), no less than 72% of the adult females selected this habitat for juvenile rearing at low densities in 1998 (Hurley, *unpublished data*). Across the intermountain west, Stoner et al. (2018) found that mule deer density increased at higher elevations in areas with higher primary productivity (as measured by NDVI), such as aspen stands (which have high NDVI). Female mule deer actively exclude conspecifics from juvenile‐rearing habitat (Mackie et al., 1998), thereby limiting competing maternal female's use of the highest quality cover types and likely reducing juvenile survival (Shallow et al., 2015). Our results on maternal age show that older female mule deer selected higher elevation, higher quality juvenile‐rearing ranges with lower predation risk from especially coyotes, consistent with our predictions (Figure 2). Part of the increased vulnerability of mule deer juveniles at low elevation may have indeed been compensatory mortality. For example, in adjacent study areas in Idaho, Shallow et al. (2015) reported a strong body condition‐dependent juvenile mortality that was driven by birth mass. Birth mass in temperate large herbivores is influenced by maternal nutritional condition the previous winter, which is affected by habitat quality the previous summer (e.g. Monteith et al., 2014). Thus, an outcome of density‐dependent responses of juvenile survival at lower than expected abundance may be explained by individual differences among female mule deer behavior, such as age, rather than by forage biomass limitation.

Despite our evidence that spatial, predator‐mediated density‐dependent mortality occurs in juvenile mule deer, there were a number of limitations to our study. First, despite our overall large sample size of *n* = 251, when considering sample sizes across years and treatments, we had in general 25–30 juveniles radiocollared per treatment–year combination. This reduces our confidence in our results for testing predator‐specific mortality rates (e.g., Figure 5) versus deer density. Nonetheless, our strongest result is a clear rejection of the classic food‐based competition hypothesis. Next, whether predator‐mediated density‐dependent neonate (0‐ to 6‐month‐old) survival will translate to mule deer population dynamics remains an open question (Bergman et al., 2015). Hurley et al. (2011) showed that juvenile survival was the key vital rate driving population growth rate of mule deer in these same study areas. While the overwinter component (i.e., from 6 to 12 months) was most influential in the variability of growth rate, the overall growth rate was still dependent on the summer neonate survival (i.e., from birth to 6 months) as juvenile recruitment is a product of both periods. The relative importance of summer versus winter survival to variation in population dynamics may vary, but was approximately equal in a nearby elk population (Eacker et al. 2017). Nonetheless, the primary driver of overwinter juvenile survival was juvenile mass at 6 months of age (Hurley et al., 2014), which was driven by forage productivity from 0 to 6 months of age (see also Bishop, White, Freddy, Watkins, & Stephenson, 2009). Thus, competition for high‐quality juvenile‐rearing habitat during summer, and the ensuing spatial density dependence it causes (Bergman et al., 2015), may set the stage for the relative importance of summer versus winter juvenile survival to population dynamics in large herbivores where competition for high‐quality juvenile‐rearing habitat occurs (e.g., roe deer, white‐tailed deer). In a wider area of Idaho across 11 GMU’s and >3,000 radiocollared adult female mule deer over 11 years, including the 2 GMU we studied here, Hurley et al. (2014) found evidence for density dependence in juvenile survival exacerbated by winter severity in about half of the populations. Although we were unable to separate out summer and overwinter survival in the populations with density‐dependent juvenile survival, our results demonstrate the key role that competition for high‐quality mule deer juvenile‐rearing ranges may have in driving overall patterns of annual juvenile survival (Bergman et al., 2015). And our results clearly show that it is not merely bottom‐up, food‐based density dependence that is the mechanism.

Competition for high‐quality habitats, space itself, was more consistent with density dependence in juvenile vital rates in our experimental study than food. Thus, space may be a key driver of population dynamics in similar settings (Morris, 2003), echoing Jeffries and Lawton (1984)’s charge to more thoroughly consider predation and enemy‐free space more in ecology. In our case study of mule deer, the mechanism by which juvenile mortality increased with population density was the increasing predation risk of juveniles to a generalist predator (coyotes) that selected low‐quality deer summer ranges that are occupied by less competitive (e.g., younger) females when density increases. These findings align with other studies in other taxa such as fish (White & Warner, 2007), birds (Andren, 1990), small mammals (Morris, 2003), and African lions (Mosser et al., 2009). Thus, the distribution of habitat quality and the number of high‐quality habitats is expected to drive the strength of density dependence, perhaps as much as food competition by itself. We thus predict that in areas with a left‐skewed distribution of habitat quality (i.e., very few sites of high quality), spatial density dependence should be the strongest and should decrease with increasing abundance of high‐quality sites. These conclusions are supported by recent spatial food‐web modeling that clearly demonstrates the critical role of space and differential spatial overlap between predators and prey in determining predictions of density‐dependent predation (Northfield et al. 2017; Schmitz et al. 2017). Ultimately, when resource abundance or forage quality directly determines spatial variation in habitat quality, food resources should be indirectly the mechanism. In our case study, though, predation risk on juveniles appeared to influence habitat quality, at least from the juvenile survival perspective, but only at high densities when competition forced mule deer females to occupy lower quality habitats. We have shown that a despotic behavior by female large herbivores could explain why density dependence in juvenile survival occurs far below the threshold when food‐based competition occurs.

## AUTHOR CONTRIBUTION

**Mark Hurley:** Conceptualization (equal); Data curation (equal); Formal analysis (equal); Funding acquisition (equal); Investigation (equal); Methodology (equal); Project administration (equal). **Mark Hebblewhite:** Conceptualization (equal); Supervision (equal); Writing‐original draft (supporting); Writing‐review & editing (equal). **Jean‐Michel Gaillard:** Conceptualization (supporting); Supervision (supporting); Writing‐review & editing (supporting).

## Supporting information

Fig S1

## Data Availability

Spatial datasets and survival datasets, along with supporting R analysis code, are available on Dryad: https://doi.org/10.5061/dryad.0cfxpnvzb
