# Supplementary material for: Competition for safe real estate, not food, drives density‐dependent juvenile survival in a large herbivore
Source: Ecol Evol. 2020 Jun 9;10(12):5464–75. doi: 10.1002/ece3.6289 (PMC7319175; doi:10.1002/ece3.6289)
Supplement: Supplementary file 1 — Fig S1 [file ECE3-10-5464-s001.docx]

**
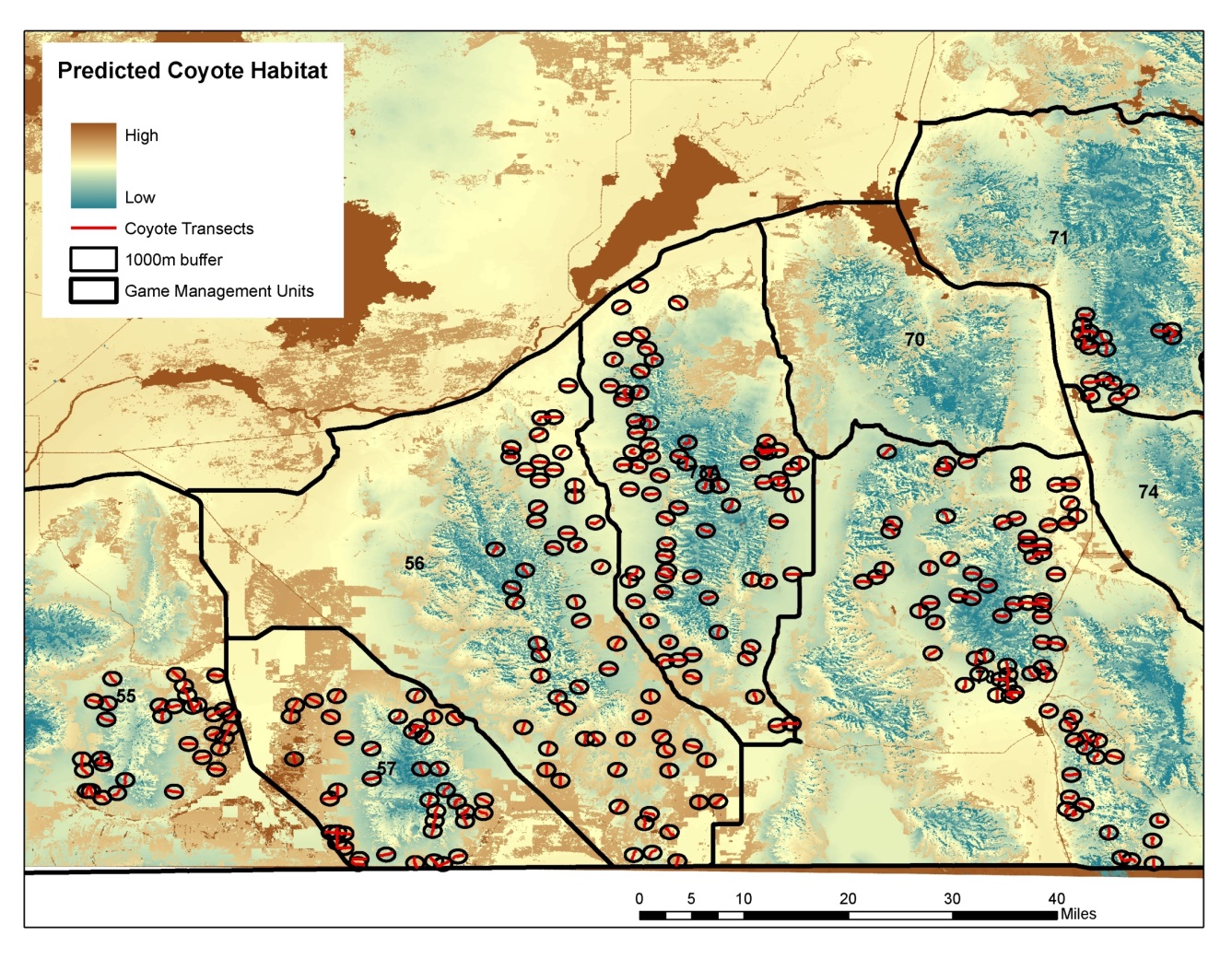
Supporting Information**

Figure S1. Spatial predictions from the resource selection probability function (RSPF) model of coyote (*Canis latrans*) predation risk for mule deer (*Odocoileus hemionus*) juvenile predation risk in southern Idaho, 1998–2002, showing the two Game Management Units 56 and 73A where juvenile mule deer were monitored. Green areas represent lower predation risk areas, but higher elevation areas (mountain ridges, etc.). The spatial distribution of coyote transects used to develop the model in a wider spatial area are depicted by black circles.
